# Resting Heart Rate Is Not a Good Predictor of a Clustered Cardiovascular Risk Score in Adolescents: The HELENA Study

**DOI:** 10.1371/journal.pone.0127530

**Published:** 2015-05-26

**Authors:** Augusto César Ferreira de Moraes, Alex Jones Flores Cassenote, Catherine Leclercq, Jean Dallongeville, Odysseas Androutsos, Katalin Török, Marcela González-Gross, Kurt Widhalm, Anthony Kafatos, Heráclito Barbosa Carvalho, Luis Alberto Moreno

**Affiliations:** 1 YCARE (Youth/Child and cARdiovascular Risk and Environmental) Research Group, School of Medicine of the University of São Paulo, São Paulo, Brazil; 2 GENUD (Growth, Exercise, Nutrition and Development) Research Group, Facultad de Ciencias de la Salud de la Universidad de Zaragoza, Zaragoza, Spain; 3 Department of Infectious and Parasitic Diseases, School of Medicine of the University of São Paulo, São Paulo, Brazil; 4 Department of Preventive Medicine, School of Medicine of the University of São Paulo, São Paulo, Brazil; 5 National Institute for Food and Nutrition Research, Rome, Italy; 6 Institut Pasteur de Lille, Lille, France; 7 Department of Nutrition & Dietetics, Harokopio University, Athens, Greece; 8 Department of Paediatrics, Medical Faculty, University of Pécs, Pécs, Hungary; 9 Faculty of Physical Activity and Sport-INEF, Technical University of Madrid, Department of Health and Human Performance, Madrid, Spain; 10 Institut für Ernährungs-und Lebensmittelwissenschaften–Humanernährung, Rheinische Friedrich-Wilhelms, Universität Bonn, Dortmund, Germany; 11 Department of Paediatrics, Private Medical University Salzburg, Salzburg, Austria; 12 Preventive Medicine & Nutrition Unit, School of Medicine, University of Crete Heraklion, Crete, Greece; Hunter College, UNITED STATES

## Abstract

**Background:**

Resting heart rate (RHR) reflects sympathetic nerve activity a significant association between RHR and all-cause and cardiovascular mortality has been reported in some epidemiologic studies.

**Methods:**

To analyze the predictive power and accuracy of RHR as a screening measure for individual and clustered cardiovascular risk in adolescents. The study comprised 769 European adolescents (376 boys) participating in the HELENA cross-sectional study (2006–2008) were included in this study. Measurements on systolic blood pressure, HOMA index, triglycerides, TC/HDL-c, VO_2_máx and the sum of four skinfolds were obtained, and a clustered cardiovascular disease (CVD) risk index was computed. The receiver operating characteristics curve was applied to calculate the power and accuracy of RHR to predict individual and clustered CVD risk factors.

**Results:**

RHR showed low accuracy for screening CVD risk factors in both sexes (range 38.5%–54.4% in boys and 45.5%–54.3% in girls). Low specificity’s (15.6%–19.7% in boys; 18.1%–20.0% in girls) were also found. Nevertheless, the sensitivities were moderate-to-high (61.4%–89.1% in boys; 72.9%–90.3% in girls).

**Conclusion:**

RHR is a poor predictor of individual CVD risk factors and of clustered CVD and the estimates based on RHR are not accurate. The use of RHR as an indicator of CVD risk in adolescents may produce a biased screening of cardiovascular health in both sexes.

## Introduction

Resting heart rate (RHR) reflects sympathetic nerve activity [1, 2], and it is an accessible clinical measurement. A significant association between resting HR and all-cause of cardiovascular mortality has been reported in some epidemiologic studies [1, 3–5]. Based on epidemiologic data and inferences from clinical trials the results showed that RHR are undesirable in terms of cardiovascular disease. However, the importance of RHR as a prognostic factor and potential therapeutic outcome has not been formally explored, and therefore, despite suggestive evidence, is not generally accepted [6].

The main metabolic cardiovascular diseases (CVD) risk factors are dyslipidemia, glucose intolerance, hypertension and obesity, which are highly prevalent in young people [7]. From a methodological perspective, the use of a clustered cardio-metabolic risk score is recommended because it can compensate for day-to-day fluctuations observed when using the single risk factors [8]. Additionally, cardio-metabolic risk factors acquired in youth, as well as their health risks, tend to persist into adulthood [9]. Therefore, identifying good predictors for cardio-metabolic risk factors is necessary to assist in the development of actions designed to improve cardio-metabolic health in young populations.

Thus, we hypothesized that RHR is a good predictive power and accuracy of RHR as screening measure for individual and clustered CVD risk in adolescents. We tested this hypothesis on the Healthy Lifestyle in Europe by Nutrition in Adolescence cross-sectional study (HELENA-CSS).

## Methods

### Study population

The HELENA-CSS aimed to describe the lifestyle and nutritional status of European adolescents. Data collection took place between October 2006 and December 2007 in the following cities: Athens and Heraklion in Greece, Dortmund in Germany, Ghent in Belgium, Lille in France, Pécs in Hungary, Rome in Italy, Stockholm in Sweden, Vienna in Austria, and Zaragoza in Spain. Further information about the study design has been published elsewhere [10, 11]. Participants were recruited at schools. To ensure that the heterogeneity of social background of the population would be represented, schools were randomly selected after stratification by school zone or district. In cases where the selected schools refused to participate, a second list of substitute schools had already been drawn up. Up to three classes from two grades were selected per school. A class was considered eligible if the participation rate was at least 70%. The general inclusion criteria for HELENA were age range of 12.5–17.5 years, not participating simultaneously in another clinical trial, and free of any acute infection lasting less than 1 week before inclusion [10].

**Fig 1:**
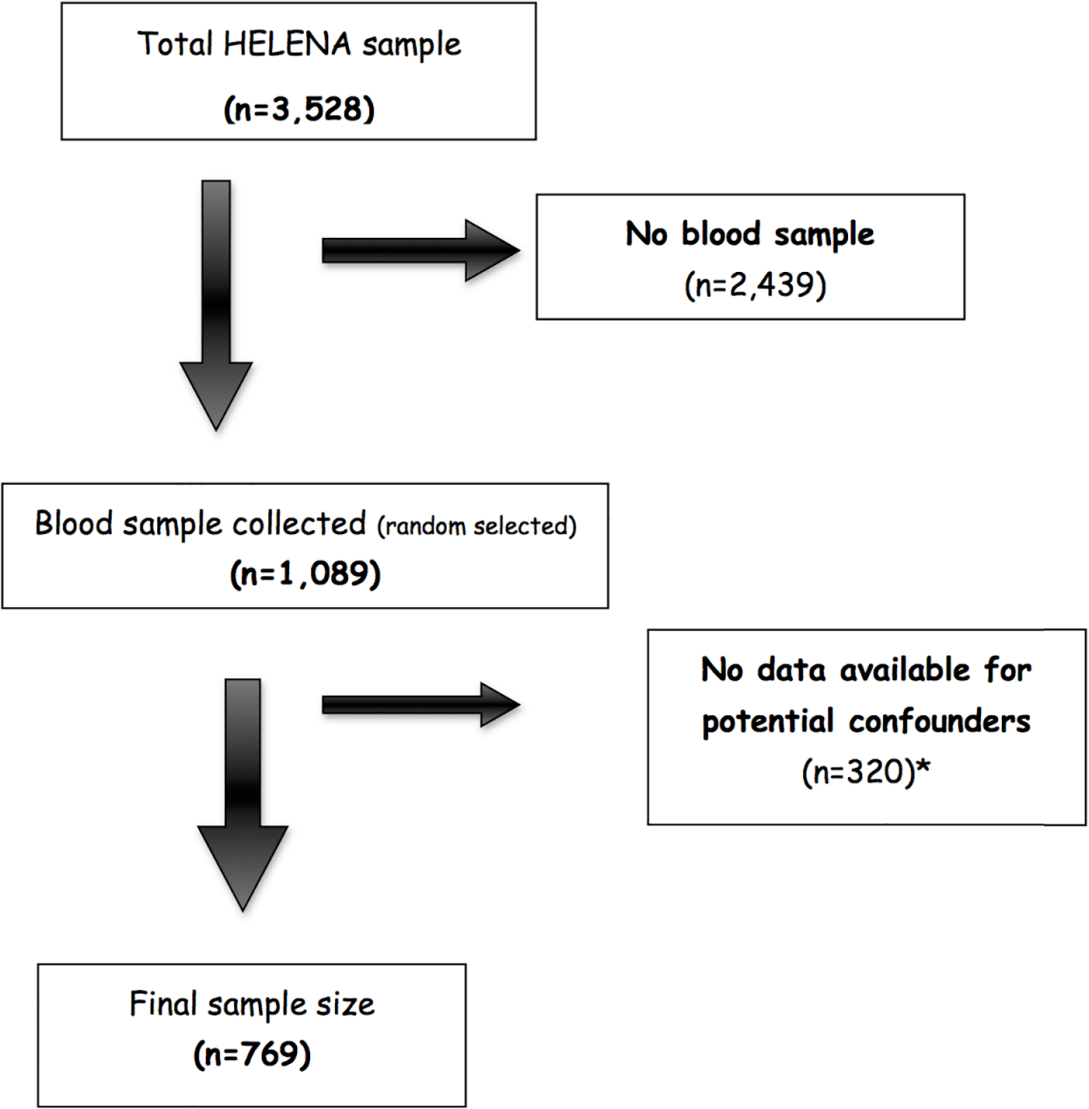
Final sample size flowchart. *Missing data for accelerometry datas.

From a sample of 3528 adolescents who met the HELENA general inclusion criteria, one third of the school classes were randomly selected in each centre for blood collection, resulting in a total of 1089 adolescents. For the purposes of the present study, adolescents with valid data for sedentary behaviour, accelerometry, cardiorespiratory fitness, total cholesterol (TC), high density lipoprotein cholesterol (HDL-c), insulin, glucose, systolic blood pressure and triceps, biceps, subscapular and supra-iliac skinfolds were finally included in the analysis (n = 769, **Fig 1**). The study sample did not differ in sex distribution, mean age, mean body mass index (BMI) and mean values of cardiorespiratory fitness from the full HELENA sample (all p>0.05).

The HELENA study was performed following the ethical guidelines of the Declaration of Helsinki 1975 (as revised in 1983). The Human Research Review Committees of all participating centers approved the study protocol and all details of this field has been published elsewhere [12]: Athens and Heraklion: Ministry of National Education and Religious Affairs; Dortmund: Research Institute of Child Nutrition, Rheinischen Friedrich-Wilhelms Universität Bonn; Ghent: Human Ethics Committees of Ghent University; Pécs: Medical Research Council Scientific; Rome: Ministry Health; Stockholm: Human Ethics Committees of Karolinska Institute; Vienna: Ministry Health; Zaragoza: Human Ethics Committees of University of Zaragoza. Written informed consent was obtained from both the adolescents and their parents.

### Resting Heart Rate (RHR)

The RHR were measured in all centers using the same type of oscillometric monitor device OMRON M6 (HEM 70001) which has been approved by the British Hypertension Society [13]. All devices were calibrated by measuring of the RHR and blood pressure following the procedure in the operations manual, we observed no significant differences between devices; measurements were taken twice (10 min apart) and the lowest value was retained, these data collection procedures have been described previously [14].

### Physical examination

Waist circumference, height, weight and four skinfold thicknesses (on the left side from biceps, triceps, subscapular, supra-iliac) were measured following a standardized protocol [15]. The definition of obesity (including overweight) was based on international BMI cutoffs proposed by Cole et al. [15] from several different countries. Systolic and diastolic blood pressure measurements by the arm blood pressure oscillometric monitor device OMRON M6 (HEM 70001) which has been approved by the British Hypertension Society [13]. Measurements were taken twice (10 min apart) and the lowest value was retained. These data collection procedures have been described previously[14].

### Cardiorespiratory fitness

Participants ran between two lines 20 m apart, keeping the pace with audio signals. The initial speed was 8.5 km/h, and each minute speed was increased by 0.5 km/h. Participants had to run in a straight line and to pivot on the lines. The test finished when subjects stopped due to fatigue or when they failed to reach the end line concurrent with the signals on two consecutive occasions. The last completed stage or half-stage was recorded. Finally, the maximal oxygen consumption (VO_2_ max) in ml/kg/min was estimated by the Leger equation (Boys and girls: VO2max = 31.025 + (3.238 x S x 3.248 x A) + (0.1536 x S x A) (A the age; S the final speed) (S = 8 + 0.5 last stage completed) [16], [17]. Physical fitness levels were described in detail elsewhere [18].

### Cardiovascular diseases risk factors

Blood samples were obtained for a third of the HELENA-CSS participants. Blood samples (24.3 ml) were collected by venipuncture at school between 8 and 10 o’clock in the morning after a 10-hour overnight fast. Centrifugation was performed at room temperature. Blood was collected in heparinized tubes, immediately placed on ice and centrifuged within 30 min (3,500 r.p.m. for 15 min) to avoid haemolysis. Immediately after centrifugation, the samples were stored and transported at 4–7°C (for a maximum of 14 h) to the central laboratory in Bonn (Germany) and stored there at—80°C until assayed. Triglycerides, TC, high-density lipoprotein cholesterol (HDL-c) and glucose were measured using enzymatic methods (Dade Behring, Schwalbach, Germany). Insulin levels were measured using an Immulite 200 analyser (DPC Bierman GmbH, Bad Nauheim, Germany). The homeostasis model assessment (HOMA) calculation was used as a measurement of insulin resistance (glycaemia X insulin/22.5) [19].

A clustered cardio-metabolic risk index was created from the following variables: systolic blood pressure, HOMA index, SBP, TG, TC/HDL-c, VO_2_max and the ∑4Skinfold. The standardized value of each variable was calculated as (value-mean)/standard deviation, separately for males and females and by 1-year age group. For variables characterized by lower metabolic risk with increasing values (HDL-c), *z*-scores were multiplied by -1. To create the cardio-metabolic risk score, all *z*-scores were summed, in which the lowest values were indicative of a better cardio-metabolic risk profile. Finally, all of those subjects at or above age- and gender- specific, Individuals with a risk score above 1 SD of the composite variable were defined as being at risk, similar to previous studies [8, 20].

### Statistical Analysis

The descriptive analyses were presented as means (quantitative variables) and percentages (qualitative variables) and confidence intervals 95% (95% CI).

All cardiovascular risk factors variables were entered as fıxed factors. Education of the mother, MVPA, waist circumference and months from menarche for girls were entered as covariates. Receiver operating characteristics (ROC) curve analysis was applied to calculate the relationship between clustered and individual cardiovascular risk factors (were used binary outcome) and RHR. ROC curve provides the whole spectrum of specifıcity/sensitivity values for all the possible cut-offs. The area under the curve (AUC) is determined from plotting sensitivity versus 1—specifıcity of a test as the threshold varies over its entire range. Taking into account the suggested cut-off points, the test can be non-informative/test equal to chance less accurate (0.5AUC<0.7); moderately accurate (0.7>AUC≤0.9); highly accurate (0.9>AUC<1.0); and perfect discriminatory tests (AUC = 1.0) [21]. In addition, ROC curve indexes of each cut-off point were calculated through the determination of positive and negative predictive values, overall misclassifıcation rate, positive and negative likelihood ratios, and Youden Index [22]. As a supplementary analysis, we assessed the association between RHR and individual and clustered cardio-metabolic risk factors by using bivariate linear regression. The magnitude of these associations was subsequently expressed in adjusted β-coefficients and their respective 95% CI. Multilevel linear regression models using mixed effects intercept were fitted to analyze the relationship between each RHR and independent variables without including co-variates [23, 24]. The context variable used was the school. Moreover, homoscedasticity was graphically assessed in all regression models to meet the criteria of this analysis.

The statistical software package Stata version 12.0 (Stata Corp., college Station, TX, USA) was used for all statistical calculations.

## Results

The proportion of boys had significantly performing physical activity the recommended amount of physical activity (≥60min/d) was higher than girls. Among CVD risk factors, males showed higher significant levels for SBP and TC/HDL, while girls had higher plasma concentrations of TC, HDL-c and triglycerides. Boys had also higher RHR than their female peers (**Table 1**).

**Table 1:** Characteristics of the study population.

Variables	Girls (n = 393)	Boys (n = 376)
	mean or % (95%CI)	mean or % (95%CI)
**Age (years)**	14.8 (14.7–14.9)	14.8 (14.7–14.9)
**Tanner Stage (%)**		
1 and 2 (pre-pubertal)	7.3 (5.0–9.6)	7.1 (4.7–9.5)
3 and 4 (pubertal)	65.6 (61.6–70.1)	64.4 (59.9–68.9)
5 (post-pubertal)	26.8 (59.9–68.9)	28.5 (24.3–32.8)
**Education mother**		
Lower education	8.4 (6.0–10.7)	8.9 (6.3–11.4)
Lower secondary education	30.3 (26.4–34.2)	27.6 (23.6–31.7)
Higher secondary education	30.7 (26.8–34.6)	29.6 (25.4–33.8)
University degree	30.6 (26.8–34.6)	33.9 (25.4–33.8)
**MVPA**		
< 60 min/d	**72.3 (67.9–76.7)**	**39.3 (33.9–44.6)**
≥ 60 min/d	**27.7 (23.3–32.1)**	**60.7 (55.4–66.1)**
**Sedentary behavior by questionnaire**		
> 4 h/d	**20.4 (18.3–22.5)**	**38.8 (36.2–41.5)**
2–4 h/d	**36.3 (33.8–38.8)**	**39.1 (36.4–41.7)**
< 2 h/d	**43.3 (40.7–45.8)**	**22.1 (19.9–24.4)**
**Months from menarche**	24.0 (22.4–25.6)	
**Height (cm)**	**162.3 (161.8–162.9)**	**169.3 (168.5–170.1)**
**Weight (kg)**	**56.7 (55.9–57.6)**	**61.0 (59.9–62.2)**
**BMI (kg/m** ^**2**^ **)**	21.5 (21.2–21.8)	21.1 (20.8–21.5)
**Obesity (%) by Cole**	**3.0 (1.3–4.6)**	**5.5 (3.1–7.8)**
**Waist circumference (cm)**	**70.6 (70.0–71.3)**	**74.4 (73.6–75.2)**
**VO2max (ml/kg/min)**	84.8 (84.4–85.2)	83.4 (83.0–83.8)
**Tryglicerides (mg/dl)**	**73.3 (70.3–76.4)**	**64.4 (61.6–67.2)**
**HDLc(mg/dl)**	**60.0 (56.1–57.9)**	**53.0 (52.1–53.9)**
**Total cholesterol(mg/dl)**	**166.9 (164.6–169.2)**	**153.8 (151.6–156.1)**
**TC/HDL-c**	2.99 (2.93–3.04)	3.02 (2.96–3.09)
**Systolic Blood Pressure (mmHg)**	**116.2 (115.3–117.1)**	**124.4 (123.1–125.8)**
**HOMA index**	2.38 (2.20–2.56)	2.28 (2.12–2.43)
**∑ Four skinfolds**	53.6 (51.5–55.8)	52.2 (49.9–54.4)
**Resting heart rate (bpm)**	**78.9 (77.8–80.00)**	**80.6 (79.3–81.8)**
**Metabolic risk (%)**	15.3 (11.8–18.9)	15.6 (11.9–19.4)

95% CI: confidence interval of 95%; BMI: body mass index; MVPA: Moderate to vigorous physical activity; HDLc = High-density lipoprotein cholesterol; TC = Total cholesterol. Significance difference (*p* <0.05) between girls and boys are in bold.

The accuracy of prediction of RHR for the six factors individual CVD risk factors and for the cluster of CVD separately by sex. For all CVD risk factors, the RHR have a high sensitivity, low specificity and accuracy (area under of curve), regardless of sex (**Table 2 and S1 Table**). The **S1 Table** presents accuracy of RHR in screening of individual and clustered cardio-metabolic risk factors separately by sex, in which all cardiovascular risk factors were adjusted by education of the mother, MVPA, waist circumference and months from menarche for girls.

**Table 2:** Accuracy of resting heart rate in screening of individual and clustered cardio-metabolic risk factors in adolescents from HELENA study.

Cardiovascular risk factors	β	Standard error	Lower CI 95%	Upper CI 95%
Clustered metabolic risk				
Male	0.807	0.172	0.489 ±0.028	0.433
Female	0.903	0.181	0.542 ±0.021	0.499
**TC/HDL-c**				
Male	0.773	0.180	0.475 ±0.026	0.423
Female	0.841	0.197	0.522 ±0.022	0.479
**VO2max**				
Male	0.855	0.185	0.520 ±0.024	0.473
Female	0.895	0.192	0.543 ±0.019	0.504
**∑ Four skin folds**				
Male	0.814	0.188	0.501 ±0.022	0.457
Female	0.838	0.196	0.517 ±0.021	0.476
**HOMA index**				
Male	0.890	0.197	0.544 ±0.023	0.498
Female	0.877	0.197	0.537 ±0.025	0.488
**Systolic Blood Pressure**				
Male	0.614	0.155	0.384 ±0.306	0.324
Female	0.728	0.181	0.455 ±0.030	0.395
**Triglycerides**				
Male	0.802	0.186	0.494 ±0.025	0.444
Female	0.897	0.200	0.538 ±0.025	0.494

CI 95% = confidence interval 95%; SE = Standard error;HDLc = High-density lipoprotein cholesterol; TC = total cholesterol.

The Table 3 presents the association RHR and individual and clustered cardio-metabolic risk factors separately by sex. We not find significant associations between RHR and CVD individual and clustered cardio-metabolic risk factors.

**Table 3:** Association between resting heart rate and individual and clustered cardio-metabolic risk factors in adolescents from HELENA study.

Cardiovascular risk factors	β	Standard error	Lower CI 95%	Upper CI 95%
Clustered metabolic risk				
Male	0.008701	0.0014625	-0.0058352	0.0115681
Female	0.001524	0.0015562	-0.0015261	0.0045741
**TC/HDL-c**				
Male	0.0095418	0.0037123	-0.0022658	0.0168178
Female	0.0029268	0.0039575	-0.0048298	0.0106834
**VO2max**				
Male	-0.0184099	0.0043384	-0.0269130	0.0099067
Female	-0.0178964	0.0040768	-0.0258867	0.0099060
**∑ Four skin folds**				
Male	0.0186863	0.003702	-0.0114294	0.0259431
Female	0.0090994	0.003885	-0.0014849	0.0167138
**HOMA index**				
Male	0.0126892	0.0034277	-0. 005971	0.0194073
Female	0.0017796	0.0040072	-0.0060743	0.0096336
**Systolic Blood Pressure**				
Male	0.0051019	0.0036015	-0.001957	0.0121608
Female	0.0038823	0.0035839	-0.003142	0.0109067
**Triglycerides**				
Male	0.0109601	0.0035406	-0.0040207	0.0178995
Female	0.0004638	0.0039252	-0.0072294	0.0081570

CI 95% = confidence interval 95%; SE = Standard Error; HDLc = High-density lipoprotein cholesterol; TC = total cholesterol.

## Discussion

This study analyzed the predictive power and accuracy of RHR as a screening measure for individual and clustered CVD risk factors in a large sample of European adolescents. The main finding was that RHR is not a good predictor of CVD risk factors in this population, regardless of sex, age and level of physical activity. Our hypothesis is biologically plausible, since the onset of these factors in adolescence is strongly associated with increased risk of CVD in adulthood [9].

Girls had lower RHR than boys (statistically significant), this difference can be partially explained by two reasons: 1) the girls have a higher VO2max and this increased aerobic capacity decreases RHR; 2) boys has a higher accumulation abdominal fat (measured by waist circumference) [25] than girls, and visceral fat has been associated with higher sympathetic activity [26, 27]. This activation is a key mechanism underlying the effect of intra-abdominal fat accumulation on the development of hypertension [28].

Recently, another study analyzed the potential effects of screening and resting heart rate (RHR) on cardiometabolic risk in adolescents [29]. They found the use of RHR to screen for alterations in glucose and triglycerides interesting but, according to the data presented, we believe that there is no evidence for this. Accuracy (AUC) for high glucose was 0.611 (95% CI 0.534–0.688) and high triglycerides, 0.618 (95% CI 0.531–0.705), both with p-values <0.05, but with low discrimination power—note the lower confidence bound in some cases is very close to 0.50 (random event). In other words, if we consider random variations within the CI bounds of AUC, determining the presence or absence of high glucose and high triglycerides will be as precise as playing a game of heads or tails. With regard to the accuracy of results, Swets [21] suggested operational cut-off points: the test can be non-informative/test equal to chance (0.5AUC<0.7); moderately accurate (0.7>AUC≤0.9); highly accurate (0.9>AUC<1.0); and perfect discriminatory tests (AUC = 1.0).

Although RHR has been recently showed to be a good predictor for CVD in adults [1], our findings do not confirm these results in adolescents. These differences may explained by the fact that the analyses carried out in adults considered as risk values into percentiles of the RHR [1, 3–5] which is intrinsically associated with the distribution of the variable within the sample. In our study, we analyzed the predictive value using a more accurate analysis (ROC curve) that the distribution in percentiles. Another important point is that the onset of CVD takes several years [30], and here only we compare with risk factors for diseases.

Fernandes et al. [31] found that higher RHR is associated with higher levels of SBP regardless of nutritional status in children, however the authors also used percentiles to classify the RHR. There is evidence that obese adolescents have higher levels of SBP [31], which might also be translated into having higher RHR. However, accurate measurement of RHR is difficult, and the biological parameter has no advantage over the use of other CVD risk factors, since RHR has been shown to have an accuracy of less than 55% for all the factors. Another important point which may explain the absence of good prediction is the fact that adolescents are in the process of biological maturation and maturation-related hormones influence the sympathetic activation [32] can be reflects vagal nerve activity that controls the RHR [1, 2].

This study adds more evidence to the existing literature about predictors of cardio-metabolic risk in adolescents. As noted, “we are biased by findings that are published and are thus blind to any studies that produce negative findings [33]”. The principal message from our study is that RHR is an important cardiovascular indicator and may play an important role in adolescents´ health, despite not being a good predictor of cardio-metabolic health during the adolescence.

Our study was developed in European adolescents, and one possible limitation is lack of generalizability of these findings to other non-European adolescent populations [34]. But we believe that this limitation is minimized by our being multicenter study conducted in 10 cities in 9 countries with different ethnic groups, and also because we studied two biological variables (cardiovascular risk and RHR), as did Doll & Hill in their major on cigarette consumption and cancer study [35], which was taken only in UK doctors and was generalized to all ethnic groups and cultures of the world, for its outcome to be a biological variable.

The strengths of this study are that samples were collected in different countries using the same methodology, appropriate statistical analysis controlling for potential confounding factors were performed as well as the analysis of the efficiency of RHR such a predictor for different individual CVD risk factors and clustered CVD. On the other hand, diverse geographic origin of the sample and multilevel analysis are some of the main strengths of our analysis.

In this study there are some limitations such as its cross-sectional design; consequently, causality cannot be established. Moreover, it has not been possible to adjust the analysis for other factors potentially associated with BP, eg. genetic, intrauterine development and inflammatory indicators.

## Conclusions

In conclusion that the RHR is a poor predictor for individual and clustered CVD risk factors. Furthermore, the estimates based on RHR are not accurate. According to our findings, the use of RHR as an indicator of cardiovascular risk in adolescents may result in a biased screening of cardiovascular health in both sexes.

## Supporting Information

S1 Dataset Dataset from HELENA Study. (XLS)

S1 File HELENA Study Group. (DOC)

S1 Table Accuracy of resting heart rate in screening of individual and clustered cardio-metabolic adjusted risk factors in adolescents from HELENA study. (DOC)
