# Supplementary material for: Resting Heart Rate Is Not a Good Predictor of a Clustered Cardiovascular Risk Score in Adolescents: The HELENA Study
Source: PLoS One. 2015 May 26;10(5):e0127530. doi: 10.1371/journal.pone.0127530 (PMC4444318; doi:10.1371/journal.pone.0127530)
Supplement: S1 File — (DOC) [file pone.0127530.s002.doc]

* HELENA Study Group

General Coordinator: LA Moreno.

Core Group members: LA Moreno, F Gottrand, S De Henauw, M González-Gross, C Gilbert.

Steering Committee: A Kafatos (President), LA Moreno, C Libersa, S De Henauw, J Sánchez, F Gottrand, M Kersting, M Sjöstrom, D Molnár, M González-Gross, J Dallongeville, C Gilbert, G Hall, L Maes, L Scalfi.

Project Manager: P Meléndez.

*Universidad de Zaragoza (Spain):*

LA Moreno, J Fleta, JA Casajús, G Rodríguez, C Tomás, MI Mesana, G Vicente-Rodríguez, A Villarroya, CM Gil, I Ara, J Revenga, C Lachen, J Fernández Alvira, G Bueno, A Lázaro, O Bueno, JF León, JMª Garagorri, M Bueno, I Iglesia, P Velasco, S Bel, L Gracia-Marco, T Mouratidou.

*Consejo Superior de Investigaciones Científicas (Spain):*

A Marcos, J Wärnberg, E Nova, S Gómez, E Ligia Díaz, J Romeo, A Veses, M Angeles Puertollano, B Zapatera, T Pozo.

*Université de Lille 2 (France):*

L Beghin, C Libersa, F Gottrand, C Iliescu, J Von Berlepsch.

*Research Institute of Child Nutrition Dortmund, Rheinische Friedrich-Wilhelms-Universität Bonn (Germany)*

M Kersting, W Sichert-Hellert, E Koeppen.

*Pécsi Tudományegyetem (University of Pécs) (Hungary):*

D Molnar, E Erhardt, K Csernus, K Török, S Bokor, Mrs. Angster, E Nagy, O Kovács, J Répasi.

*University of Crete School of Medicine (Greece):*

A Kafatos, C Codrington, M Plada, A Papadaki, K Sarri, A Viskadourou, C Hatzis, M Kiriakakis, G Tsibinos, C Vardavas, M Sbokos, E Protoyeraki, M Fasoulaki.

*Institut für Ernährungs- und Lebensmittelwissenschaften – Ernährungphysiologie. Rheinische Friedrich Wilhelms Universität (Germany):*

P Stehle, K Pietrzik, M González-Gross, C Breidenassel, A Spinneker, J Al-Tahan, M Segoviano, A Berchtold, C Bierschbach, E Blatzheim, A Schuch, P Pickert.

*University of Granada (Spain)*

MJ Castillo, A Gutiérrez, FB Ortega, JR Ruiz, EG Artero, V España, D Jiménez-Pavón, P Chillón, C Sánchez-Muñoz, M Cuenca.

*Istituto Nazionale di Ricerca per gli Alimenti e la Nutrizione (Italy)*

D Arcella, E Azzini, E Barrison, N Bevilacqua, P Buonocore, G Catasta, L Censi, D Ciarapica, P D’Acapito, M Ferrari, M Galfo, C Le Donne, C Leclercq, G Maiani, B Mauro, L Mistura, A Pasquali, R Piccinelli, A Polito, R Spada, S Sette, M Zaccaria.

*University of Napoli "Federico II" Dept of Food Science (Italy)*

L Scalfi, P Vitaglione, C Montagnese.

*Ghent University (Belgium)*

I De Bourdeaudhuij, S De Henauw, T De Vriendt, L Maes, C Matthys, C Vereecken, M de Maeyer, C Ottevaere, I Huybrechts.

*Medical University of Vienna (Austria)*

K Widhalm, K Phillipp, S Dietrich, B Kubelka, M Boriss-Riedl.

*Harokopio University (Greece)*

Y Manios, E Grammatikaki, Z Bouloubasi, T Louisa Cook, S Eleutheriou, O Consta, G Moschonis, I Katsaroli, G Kraniou, S Papoutsou, D Keke, I Petraki, E Bellou, S Tanagra, K Kallianoti, D Argyropoulou, K Kondaki, S Tsikrika, C Karaiskos.

*Institut Pasteur de Lille (France)*

J Dallongeville, A Meirhaeghe.

*Karolinska Institutet (Sweden)*

M Sjöström, J Ruiz, FB Ortega, M Hagströmer, L Hallström, E Patterson, L Kwak, J Wärnberg, N Rizzo, A Hurtig Wennlöf.

*Asociación de Investigación de la Industria Agroalimentaria (Spain)*

J Sánchez-Molero, E Picó, M Navarro, B Viadel, JE Carreres, G Merino, R Sanjuán, M Lorente, MJ Sánchez, S Castelló.

*Campden BRI (United Kingdom)*

C Gilbert, S Thomas, E Allchurch, P Burguess.

*SIK - Institutet foer Livsmedel och Bioteknik (Sweden)*

G Hall, A Astrom, A Sverkén, A Broberg.

*Meurice Recherche & Development asbl (Belgium)*

A Masson, C Lehoux, P Brabant, P Pate, L Fontaine.

*Campden & Chorleywood Food Development Institute (Hungary)*

A Sebok, T Kuti, A Hegyi.

*Productos Aditivos SA (Spain)*

C Maldonado, A Llorente.

*Cárnicas Serrano SL (Spain)*

E García.

*Cederroth International AB (Sweden)*

H von Fircks, M Lilja Hallberg, M Messerer

*Lantmännen Food R&D (Sweden)*

M Larsson, H Fredriksson, V Adamsson, I Börjesson.

*European Food Information Council (Belgium)*

L Fernández, L Smillie, J Wills.

*Universidad Politécnica de Madrid (Spain)*

Universidad Politécnica de Madrid (Spain): Marcela González-Gross, Jara Valtueña, David Jiménez-Pavón, Ulrike Albers, Raquel Pedrero, Gonzalo Palacios, Agustín Meléndez, Pedro J. Benito, Juan José Gómez Lorente, David Cañada, Alejandro Urzanqui, Juan Carlos Ortiz, Francisco Fuentes, Rosa María Torres, Paloma Navarro.
