# Supplementary material for: Resting Heart Rate Is Not a Good Predictor of a Clustered Cardiovascular Risk Score in Adolescents: The HELENA Study
Source: PLoS One. 2015 May 26;10(5):e0127530. doi: 10.1371/journal.pone.0127530 (PMC4444318; doi:10.1371/journal.pone.0127530)
Supplement: S1 Table — (DOC) [file pone.0127530.s003.doc]

**S1 Table.**

Unadjusted ROC curve analysis of resting heart rate in screening of individual and clustered cardio-metabolic risk factors in adolescents from HELENA study.

| **Cardiovascular risk factors** | **Sensitivity** | **Specificity** | **Area Under the Curve** ±SE | **CI 95%** | |
| --- | --- | --- | --- | --- | --- |
|  |  |  |  | **Lower** | **Upper** |
| **Clustered metabolic risk** |  |  |  |  |  |
| Male | 0.807 | 0.172 | 0.489 ±0.028 | 0.433 | 0.545 |
| Female | 0.903 | 0.181 | 0.542 ±0.021 | 0.499 | 0.584 |
| **TC/HDL-c** |  |  |  |  |  |
| Male | 0.773 | 0.180 | 0.475 ±0.026 | 0.423 | 0.526 |
| Female | 0.841 | 0.197 | 0.522 ±0.022 | 0.479 | 0.566 |
| **VO2max** |  |  |  |  |  |
| Male | 0.855 | 0.185 | 0.520 ±0.024 | 0.473 | 0.566 |
| Female | 0.895 | 0.192 | 0.543 ±0.019 | 0.504 | 0.581 |
| **∑ Four skin folds** |  |  |  |  |  |
| Male | 0.814 | 0.188 | 0.501 ±0.022 | 0.457 | 0.544 |
| Female | 0.838 | 0.196 | 0.517 ±0.021 | 0.476 | 0.559 |
| **HOMA index** |  |  |  |  |  |
| Male | 0.890 | 0.197 | 0.544 ±0.023 | 0.498 | 0.566 |
| Female | 0.877 | 0.197 | 0.537 ±0.025 | 0.488 | 0.587 |
| **Systolic Blood Pressure** |  |  |  |  |  |
| Male | 0.614 | 0.155 | 0.384 ±0.306 | 0.324 | 0.444 |
| Female | 0.728 | 0.181 | 0.455 ±0.030 | 0.395 | 0.514 |
| **Triglycerides** |  |  |  |  |  |
| Male | 0.802 | 0.186 | 0.494 ±0.025 | 0.444 | 0.544 |
| Female | 0.897 | 0.200 | 0.538 ±0.025 | 0.494 | 0.582 |

CI 95% = confidence interval 95%; SE = Standard Error; HDLc = High-density lipoprotein cholesterol; TC = total cholesterol.

**Supplementary file 2.**

Association between resting heart rate and individual and clustered cardio-metabolic risk factors in adolescents from HELENA study.

| **Cardiovascular risk factors** | **β** | **Standard error** | **CI 95%** | |
| --- | --- | --- | --- | --- |
|  |  |  | **Lower** | **Upper** |
| **Clustered metabolic risk** |  |  |  |  |
| Male | 0.008701 | 0.0014625 | -0.0058352 | 0.0115681 |
| Female | 0.001524 | 0.0015562 | -0.0015261 | 0.0045741 |
| **TC/HDL-c** |  |  |  |  |
| Male | 0.0095418 | 0.0037123 | -0.0022658 | 0.0168178 |
| Female | 0.0029268 | 0.0039575 | -0.0048298 | 0.0106834 |
| **VO2max** |  |  |  |  |
| Male | -0.0184099 | 0.0043384 | -0.0269130 | 0.0099067 |
| Female | -0.0178964 | 0.0040768 | -0.0258867 | 0.0099060 |
| **∑ Four skin folds** |  |  |  |  |
| Male | 0.0186863 | 0.003702 | -0.0114294 | 0.0259431 |
| Female | 0.0090994 | 0.003885 | -0.0014849 | 0.0167138 |
| **HOMA index** |  |  |  |  |
| Male | 0.0126892 | 0.0034277 | -0. 005971 | 0.0194073 |
| Female | 0.0017796 | 0.0040072 | -0.0060743 | 0.0096336 |
| **Systolic Blood Pressure** |  |  |  |  |
| Male | 0.0051019 | 0.0036015 | -0.001957 | 0.0121608 |
| Female | 0.0038823 | 0.0035839 | -0.003142 | 0.0109067 |
| **Triglycerides** |  |  |  |  |
| Male | 0.0109601 | 0.0035406 | -0.0040207 | 0.0178995 |
| Female | 0.0004638 | 0.0039252 | -0.0072294 | 0.008157 |

CI 95% = confidence interval 95%; SE = Standard Error; HDLc = High-density lipoprotein cholesterol; TC = total cholesterol.
